# Long-Term Outcomes of Bariatric Surgery in Iraq: A Call for More Research

**DOI:** 10.7759/cureus.73554

**Published:** 2024-11-12

**Authors:** Aqeel Shakir Mahmood, Dhafer Mohammed Jasim, Adel Hashim Jebur, Sabah N Abdulraheem, Mustafa Ismail

**Affiliations:** 1 Department of Surgery, College of Medicine, University of Baghdad, Baghdad, IRQ; 2 Department of Surgery, The Private Nursing House Hospital, Medical City Complex, Baghdad, IRQ; 3 Department of Surgery, Baghdad Teaching Hospital, Baghdad, IRQ

**Keywords:** bariatric surgery, iraq, long-term outcomes, obesity, research

## Abstract

Bariatric surgery offers significant benefits of weight loss and improvement of comorbidities for morbidly obese patients. Significant and long-term studies around the world have shown that weight reduction is durable, with improvement in the quality of life and resolution of comorbidities after surgery. In Iraq, preliminary studies are encouraging for short-term outcomes, but there is a definite lack of long-term data. This editorial identifies a deficiency in long-term data. It points out that comprehensive longitudinal research is required in Iraq to establish whether the accruable benefits of bariatric surgery are sustainable and to monitor possible complications. Establishing robust, long-term studies will aid in improving patient outcomes, healthcare policies, and contribute valuable data to global research. Collaboration among healthcare providers, researchers, and policymakers is crucial to addressing this gap and enhancing the overall healthcare landscape in Iraq.

## Editorial

Background

There has been a broad acknowledgment that bariatric surgery is an effective intervention in preventing morbid obesity, leading to substantial weight loss and a significant improvement in obesity-related comorbidities such as type 2 diabetes, hypertension, and dyslipidemia in patients. International studies in different countries have proven these benefits by underlining the role that such a procedure is supposed to play in the comprehensive management of obesity [1, 2]. Despite this global evidence, there is a discernible lack of research focusing on the long-term outcomes of bariatric surgery in Iraq. While initial studies indicate promising short-term results, the absence of extensive longitudinal data hampers a thorough understanding of the sustainability of these benefits and the potential long-term complications specific to the Iraqi population.

Given the unique healthcare challenges in Iraq, including limited resources and a healthcare system still recovering from years of conflict, it is crucial to establish robust, long-term studies. Such research would not only fill the existing knowledge gap but also inform better healthcare policies and improve patient care strategies. This editorial concludes by showing the present status of bariatric surgery research in Iraq, describing different study findings and establishing the importance of long-term comprehensive research.

Global perspective on bariatric surgery outcomes

Globally, bariatric surgery has been associated with significant long-term weight loss and improvement in comorbidities such as type 2 diabetes, hypertension, and dyslipidemia. For instance, a study by Courcoulas et al. (2014) [1] summarized findings from a National Institutes of Health symposium, highlighting durable weight loss and improvement in diabetes and lipid profiles five years post-surgery. Additionally, a systematic review by Driscoll et al. (2016) [2] confirmed significant enhancements in health-related quality of life over a long-term period.

Resource limitation, inadequate access to specialized care, and challenges in the follow-up of patients for long periods have been some major limiting factors against better outcomes in the case of bariatric surgery in Iraq. These may herald higher complication rates and poor ability for sustained postoperative care; thus, region-specific studies to understand and address these unique challenges are required.

Bariatric surgery in Iraq

The rapidly changing face of bariatric practices in Iraq is evidenced by a swift upsurge in the number of cases being recognized and surgeries performed around the entire country. This increase in recognition resulted from the increased recognition that bariatric surgery is a suitable form of treatment effective against morbidity, evidenced by the ongoing upsurge in cases of obesity in Iraq. The comparative study by Mukhtar (2013) [3] sheds light on the immediate benefits of these procedures. Conducted in a Baghdad hospital, this study evaluated the short-term outcomes of three bariatric procedures: laparoscopic gastric banding (LAGB), laparoscopic sleeve gastrectomy (LSG), and laparoscopic Roux-en-Y gastric bypass (LRYGB). The findings were promising, with all procedures resulting in significant weight reduction and improvement in medical comorbidities within the first three months post-surgery. Specifically, the study reported an average weight loss of 10.97 kg, 12.34 kg, and 12.33 kg one month post-surgery for LAGB, LSG, and LRYGB, respectively. At three months, the average weight loss increased to 18.81 kg, 22.48 kg, and 24.33 kg, demonstrating substantial short-term efficacy. However, despite these encouraging short-term results, the general lack of long-term follow-up data was clearly underscored in the study, which is paramount to obtaining a whole idea of the sustainability of these benefits. Long-term data are essential for assessment not only of the persistence of weight loss but also of the resolution of comorbidities like diabetes, hypertension, and dyslipidemia. Understanding the long-term complications, such as nutritional deficits and surgical ones, is necessary for developing strategies related to patient management after surgery.

The lack of such data in Iraq underscores the urgent need for longitudinal studies to assess the enduring impact of bariatric surgery on patient health. These studies would provide invaluable insights, guiding healthcare policies and clinical practices to enhance the overall outcomes for bariatric patients in Iraq. By addressing this gap, Iraq can contribute to the global body of research and improve the quality of care for its population.

The need for research

The scarcity of long-term data in Iraq represents a significant barrier to fully understanding the impact of bariatric surgery on the Iraqi population. Mukhtar's study, while insightful in highlighting the short-term benefits of bariatric procedures, underscores a critical gap in research: the lack of sustained follow-up data. This limitation hinders the ability to evaluate the long-term effectiveness of these surgeries, particularly in maintaining weight loss and managing associated comorbidities such as diabetes, hypertension, and dyslipidemia. The absence of this data also makes it difficult to identify and address potential long-term complications, such as nutritional deficiencies, which are known to occur in bariatric patients due to the malabsorptive nature of some procedures [3].

O’Brien et al. (2013) [4] conducted a comprehensive study on the long-term outcomes of laparoscopic adjustable gastric banding, emphasizing the necessity of continuous follow-up to ensure the durability of weight loss and to monitor for complications. Their research demonstrated that without long-term data, it is challenging to develop effective management strategies for patients post-surgery, particularly in the context of revisional procedures and the evolution of bariatric techniques. Similar research conducted in Australia has highlighted the importance of long-term studies, which have shown that tracking weight trajectories and health outcomes over several years is crucial for understanding the full impact of bariatric surgery. These studies reveal that while initial weight loss can be significant, the sustainability of these results varies, and long-term monitoring is essential for managing any adverse outcomes effectively [5].

We propose a framework of study to be conducted with regard to the success rate of bariatric surgery in Iraq, with follow-up ranging from five to 10 years. A number of key measures would include weight loss, as determined by change in body mass index; resolution of comorbid conditions, such as diabetes and hypertension; perceived quality of life; nutritional deficiencies; and postoperative complications. Evaluations would be conducted at six months, one year, and yearly thereafter using standardized assessments such as the Short Form Survey Instrument (SF-36), developed by RAND Healthcare, for quality of life and biochemical markers of nutritional health.

The Iraqi context

The unique healthcare challenges in Iraq necessitate the implementation of longitudinal studies that are specifically tailored to the local context. Iraq’s healthcare system, which is still recovering from years of conflict, faces several obstacles, including limited resources, infrastructure deficits, and a shortage of healthcare professionals. These challenges complicate the delivery of consistent and high-quality post-surgical care, which is essential for the long-term success of bariatric surgery. Longitudinal studies in Iraq should be designed with a robust methodology, ensuring a follow-up period of at least five to 10 years. This duration is crucial to adequately track not only weight maintenance but also the resolution of comorbidities, improvements in quality of life, and the emergence of any long-term complications [3].

Conducting such comprehensive studies in Iraq will require a coordinated effort involving healthcare providers, researchers, and policymakers. The collaboration between these stakeholders is essential to ensure that data collection and analysis are thorough and that the studies are conducted in a manner that accounts for the specific challenges of the Iraqi healthcare system. Furthermore, these studies should include a diverse range of participants to ensure that the findings are representative of the broader Iraqi population. By investing in long-term research, Iraq can build a body of evidence that not only informs local clinical practices and health policies but also contributes to the global understanding of bariatric surgery outcomes in different cultural and healthcare settings. This research will be invaluable in improving the quality of care for bariatric patients in Iraq and ensuring that the benefits of surgery are sustained over the long term.

Conclusion

Bariatric surgery holds significant promise for patients with morbid obesity in Iraq, as evidenced by short-term outcomes. However, to truly understand its long-term impact, there is an urgent need for more extensive and longitudinal research. By prioritizing this research, Iraq can not only improve patient outcomes but also contribute valuable data to the global understanding of bariatric surgery. Investing in such research will ultimately lead to better health policies and patient care strategies, benefiting the broader population.

Research on bariatric surgery can potentially enrich the world with information emanating from Iraq, which faces many unique challenges in the form of resource limitations and infrastructural constraints. It is in this light that Iraq will implicitly be able to contribute to such a diverse understanding of the long-term outcomes of bariatric surgery, especially in resource-poor healthcare regions, by presenting longitudinal data for a population with different socioeconomic factors. This might provide a way to harness the information for globally informed strategies in comorbidity management, improve patient care for similar contexts, and develop tailored protocols for postoperative care that could be adaptable in a variety of healthcare settings around the world.
